# LEE011 and ruxolitinib: a synergistic drug combination for natural killer/T-cell lymphoma (NKTCL)

**DOI:** 10.18632/oncotarget.25835

**Published:** 2018-08-07

**Authors:** Yan Ting Hee, Junli Yan, Dean Nizetic, Wee-Joo Chng

**Affiliations:** ^1^ Lee Kong Chian School of Medicine, Nanyang Technological University, Singapore; ^2^ Cancer Science Institute of Singapore, National University of Singapore, Singapore; ^3^ The Blizard Institute, Barts and The London School of Medicine, Queen Mary **University** of London, London, UK; ^4^ Department of Haematology-Oncology, National University Cancer Institute of Singapore, National University Health System, Singapore; ^5^ Department of Medicine, Yong Loo Lin School of Medicine, National University of Singapore, Singapore

**Keywords:** NKTCL, LEE011, ruxolitinib, drug combination, lymphoma

## Abstract

Natural killer/T-cell lymphoma (NKTCL) is an aggressive non-Hodgkin lymphoma that has been facing limited success with conventional treatments, urging for the discovery of alternative strategies. Recent studies including ours have revealed that EZH2 and JAK-STAT signalling pathways are key contributors to NKTCL pathogenesis. In particular, we found that EZH2 is overexpressed and directly transcriptionally activates the *CCND1* gene to confer growth advantage. *CCND1* codes for cyclin D1, which complexes with CDK4/6 to promote G1 to S phase transition. Therefore in this study we investigated whether inhibiting both JAK1/2 and CDK4/6, using LEE011 and ruxolitinib respectively is effective in NKTL. We first demonstrate that separate LEE011 and ruxolitinib treatment is sufficient to cause growth inhibition of NKTCL cells. More importantly, we found that there is synergistic growth inhibitory effects on NKTCL cells with combination treatment of LEE011 and ruxolitinib. The results obtained shows that the targeting of both CDK4/6 and JAK1/2 are promising to develop better treatment alternatives for NKTCL.

## INTRODUCTION

Natural killer/T-cell lymphoma (NKTCL) is an aggressive type of non-Hodgkin lymphoma characterised by a clonal proliferation of NK or T-cells, and patients often have poor survival rates [1, 2]. To date, treatments for NKTCL are still completely reliant on radiotherapy, chemotherapy or a combination of both, but these have been met with limited success with low complete response rates [1, 3, 4]. There is thus a compelling need to develop alternative strategies for NKTCL.

Recent studies have reported enriched and activated (phosphorylated) STAT3 protein in NKTCL [5–8]. The JAK-STAT pathway is essential for blood cells to respond to extracellular cytokines via cytokine receptors for proliferation and growth [9]. Signals from these receptors are often propagated through JAKs that can ultimately lead to the increase in cancer cells’ survival and proliferative indices via a change in transcription profile through STATs [10, 11]. The activated STAT3 status was subsequently found to be due to STAT3 activating mutations and/or the loss of receptor-type tyrosine-protein phosphatase κ [7, 12, 13] and could be brought down to normal phosphorylated STAT3 levels upon JAK2 inhibition [7, 13]. These findings thus suggest that the STAT3 pathway could be a promising target for alternative NKTCL treatment.

Additionally, our previous studies show that the epigenetic writer EZH2 is overexpressed in NKTCL [6, 14]. EZH2 functions in the Polycomb repressive complex 2 (PRC2) along with EED, SUZ12 and RbAp46/48 to trimethylate histone H3 lysine 27 (H3K27me3), a mark often associated with gene repression [15–17]. Contrary to its epigenetic repression function, we have found that EZH2 directly transcriptionally activates the *CCND1* gene independent of its methyltransferase activity in NKTCL [14]. The *CCND1* gene codes for cyclin D1, which when complexed with CDK4/6, promotes cell cycle progression from G1 to S phase. The overexpression of EZH2 thus likely conferred growth advantage to NKTCL cells by a corresponding upregulation of cyclin D1. Indeed, high levels of *CCND1* transcript has been reported in NKTCL cell lines and upregulated cyclin D1 protein levels has been linked to poor prognosis and decreased survival in NKTCL patients [5, 18]. Hence, targeting CDK4/6 that is downstream of EZH2 could be promising for the treatment of NKTCL by inhibiting cell cycle progression.

Besides being essential to the pathogenesis of NKTCL, the JAK-STAT and EZH2-CDK4/6 pathways were noted to be upstream and downstream in the signalling pathway for cell growth respectively. As such, inhibiting them simultaneously should bring about a more robust and enhanced growth inhibition effect. Here, ruxolitinib and LEE011 (ribociclib) which targets JAK1/2 and CDK4/6 [19, 20] respectively were tested against several NKTCL cell lines. Since these two drugs have successfully reached clinical trials, it is hoped that they will show promising results in NKTCL as well. By cell viability assay, it was clearly shown that though these two drugs are able to work individually to inhibit growth of NKTCL cells, a far greater growth inhibition could be achieved when they are used in combination. Changes in apoptotic and proliferative markers and cell cycle analysis further support this observation. These findings thus strongly provide a basis for a promising alternative treatment for NKTCL patients.

## RESULTS

### Growth inhibition on NKTCL cell lines was achieved with independent CDK4/6 and JAK1/2 inhibition

**Figure 1:**
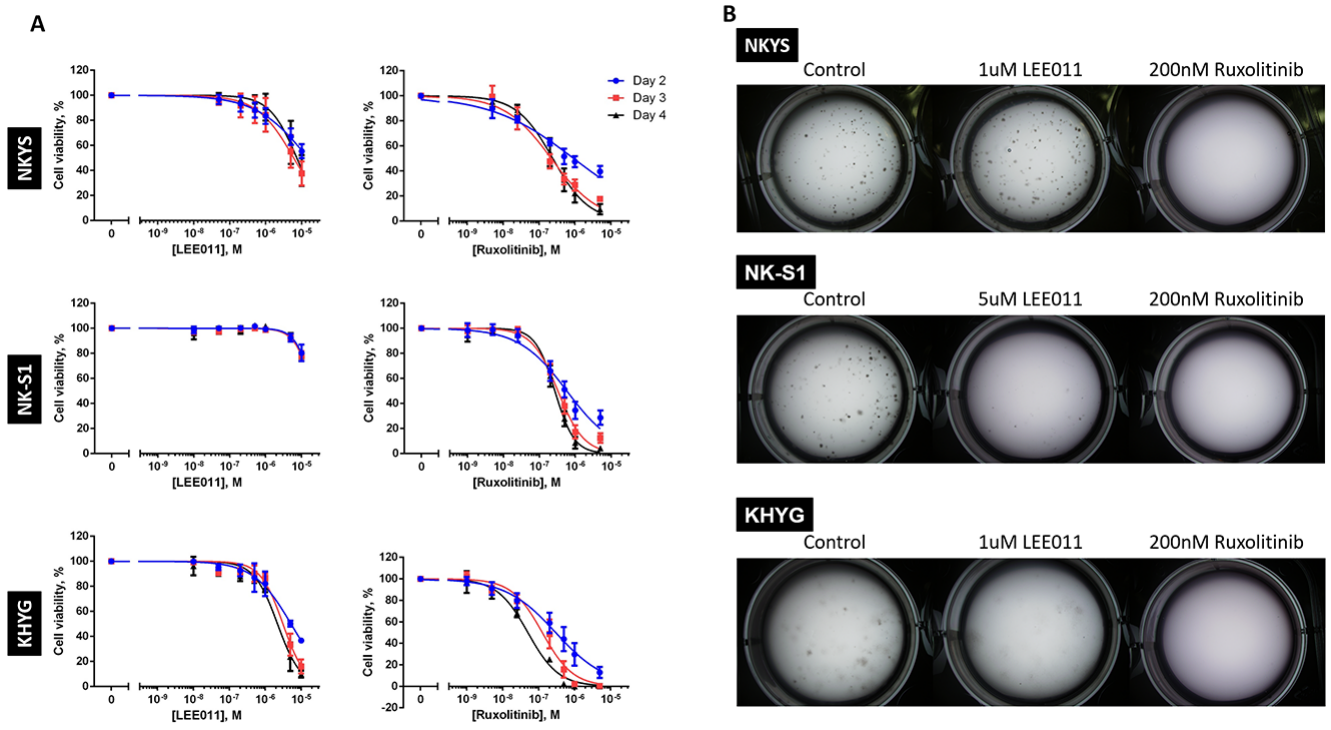
LEE011 and ruxolitinib inhibits growth in NKTCL cell lines **(A)** Cell viability assay showed growth inhibition followed after independent LEE011 and ruxolitinib treatment. NKTCL cell lines were separately treated with LEE011 and ruxolitinib and cell viabilities were assessed at Day 2, 3 and 4. In each experiment, triplicate values were averaged and treated wells were normalised against control wells. Data is expressed as mean ± SEM from three independent experiments. The IC50 curves were plotted based on the variable-slope (four-parameter logistic model), fitted using the least squares model. **(B)** Clonogenic assay of single LEE011- and ruxolitinib-induced growth inhibition. Cell lines were treated with LEE011 or ruxolitinib at concentrations roughly corresponding to the respective IC50 values determined and checked for colony formation after two weeks (n = 3, ^*^ p<0.05, ^**^ p<0.01, ^***^ p<0.001, one-way ANOVA test).

To evaluate growth inhibitory efficacy of CDK4/6 and JAK1/2 inhibition separately, the NKTCL cell lines were treated to a range of LEE011 and ruxolitinib concentrations. Cells were treated over a period of four days, with re-treatment on the second day to account for loss of drug stability over extended time in the incubator. Cell viability was assessed on Day 2, 3 and 4 of treatment. Growth inhibition was achieved with the two drugs independently across almost all the NKTCL cell lines tested as seen in the drop in cell viability as a function of drug concentration (Figure 1A). From the IC50 curves obtained, the IC50 values of LEE011 and ruxolitinib were then determined for each of the NKTCL cell lines (Table 1).

**Table 1:** IC50 values of LEE011 and ruxolitinib in all NKTCL cell lines tested

Day	NKYS		NK-S1		KHYG	
	LEE011/μM	Ruxolitinib/μM	LEE011/μM	Ruxolitinib/μM	LEE011/μM	Ruxolitinib/μM
2	15.0	0.920	23.7	0.581	5.21	0.298
3	6.00	0.219	20.8	0.327	3.11	0.126
4	7.32	0.241	19.4	0.269	2.19	0.0501

These values were obtained from the plots in Figure 1.

While the IC50 values of LEE011 fell within the 300 nM to 3.2 uM that has been determined to be sensitive in neuroblastoma and T-ALL cell lines respectively [21, 22], the concentrations were nonetheless in the micromolar range. To confirm if the decrease in cell viability resulted from LEE011 is due to inhibition of its intended targets, we thus performed knockdown (KD) with CDK4/6 siRNA which was compared to negative control siRNA. Decrease in cell viability followed after CDK4/6 KD (Supplementary Figure 1A). We also noted the extent of the drop in cell viability seemed to correlate with CDK4/6 KD efficiency (Supplementary Figure 1B) as the less efficient CDK6 KD in KHYG is reflected in the least drop in cell viability. This thus suggests that the effect of LEE011 is likely due to CDK4/6 inhibition.

Growth inhibitory efficacies of the two agents were also then demonstrated in clonogenic assays which served to detect cells that have survived and retained progeny-producing capacity following treatment [23]. Each cell line was treated with LEE011 and ruxolitinib separately at roughly the IC50 values obtained (Table 1) and was left to grow in the incubator for two weeks. In accordance to the cell viability assays, lesser number of colonies were observed in treated wells as compared to control wells across most NKTCL cell lines tested (Figure 1B). These results thus demonstrated that LEE011 and ruxolitinib are able to inhibit NKTCL cell growth individually.

### LEE011 and ruxolitinib-induced growth inhibition is mediated through blocking cell cycle progression and STAT signalling respectively

**Figure 2:**
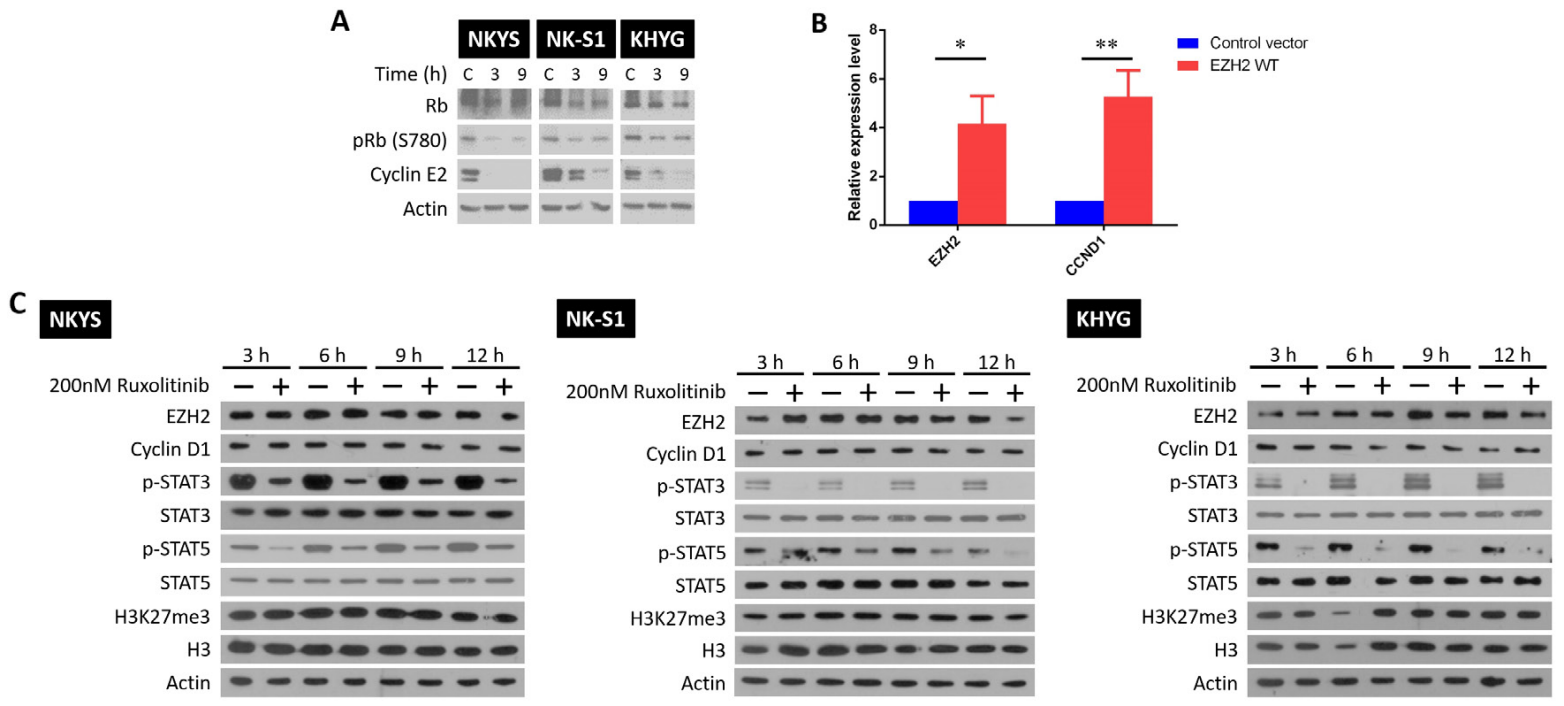
LEE011 and ruxolitinib inhibit growth by blocking cell cycle progression and STAT signalling respectively **(A)** Western blot showing that LEE011 blocks CDK4/6 phosphorylation of Rb and the subsequent expression of cyclin E2. Cells were treated with their respective LEE011 IC50 concentrations and harvested 3 and 9 h after. **(B)** RT-qPCR results of mRNA harvested from NKYS cells that were either transfected with control and EZH2 WT plasmids after 24 h. Relative fold change of *EZH2* and *CCND1* mRNA levels were obtained by normalising to control cells. Differences were determined to be statistically significant (n = 3, ^*^ p<0.05, ^**^ p< 0.01, one-tailed student’s *t*-test). **(C)** NKYS, NK-S1 and KHYG cells were treated with 200 nM ruxolitinib and harvested after 3, 6, 9 and 12 h later. Blots shown for each cell line are representative blots from the same experiment, among two other independent biological replicates.

Next, we studied how growth inhibition is achieved by LEE011 and ruxolitinib independently in NKTCL. For LEE011, it likely prevents progression into S phase by targeting CDK4/6 and inhibiting complex formation with cyclin D1 [19]. Indeed, upon LEE011 treatment, decreased cyclin E2 and phosphorylated retinoblastoma (Rb) levels were noted (Figure 2A). The mechanism of action by ruxolitinib, however, is not as clear due to JAK1/2 involvement in other pathways besides STAT signalling. In particular, JAK2 phosphorylation of EZH2 at tyrosine 641 is able to promote EZH2 degradation via the SCF^β-TrCP^ E3 ubiquitin ligase pathway in B cell lymphoma [24]. In addition, JAK3 phosphorylation of EZH2 was found to mediate EZH2 switch to non-canonical function [25]. It would thus be interesting to see if similar JAK2-mediated EZH2 degradation exists in NKTCL or that EZH2 switch to non-canonical function can be mediated by JAK1/2 as well. Gaining insights into the ruxolitinib effects on EZH2 activity is essential given EZH2’s key role in NKTCL oncogenesis [14].

It was first confirmed if EZH2 was able to transcriptionally activate *CCND1* gene expression. For this, control plasmids or plasmids containing EZH2 wild-type (WT) sequence were transfected into NKYS, followed by measuring changes in *CCND1* mRNA levels (Figure 2B). As seen, overexpression of EZH2 WT led to a corresponding increase in *CCND1* mRNA levels, affirming EZH2’s non-canonical function as transcriptional activator. This result provided proof that the non-canonical function of EZH2 can be monitored by cyclin D1 protein levels. Next, the three NKTCL cell lines were subjected to single ruxolitinib treatment at a concentration close to the IC50 values and changes in protein levels were studied. If a JAK2-mediated EZH2 degradation exists, an increase in the levels of EZH2, cyclin D1 and the H3K27me3 mark that EZH2 catalyses would be expected upon JAK2 inhibition by ruxolitinib. Likewise, if JAK1/2 phosphorylation is able to cause a functional switch in EZH2 activity, an increase in H3K27me3 levels should follow after ruxolitinib treatment as EZH2 would be redirected back to catalyse the deposition of the H3K27me3 mark. None of these trends were seen in all the NKTCL cell lines tested (Figure 2C), indicating that JAK1/2 regulation on EZH2’s activity is absent in NKTCL. It is affirmative that a successful JAK1/2 inhibition by ruxolitinib was achieved as seen by the decrease in p-STAT3/5 protein levels between control and treated cells. To note, KHYG was revealed to have a far lower STAT3/5 basal phosphorylation level than the other two cell lines (Supplementary Figure 2), which might account for the lower ruxolitinib IC50 concentration. Nonetheless, LEE011 and ruxolitinib worked as intended to cause growth inhibition in NKTCL.

### Combination treatment of LEE011 and ruxolitinib induced synergistic growth inhibitory effects

**Figure 3:**
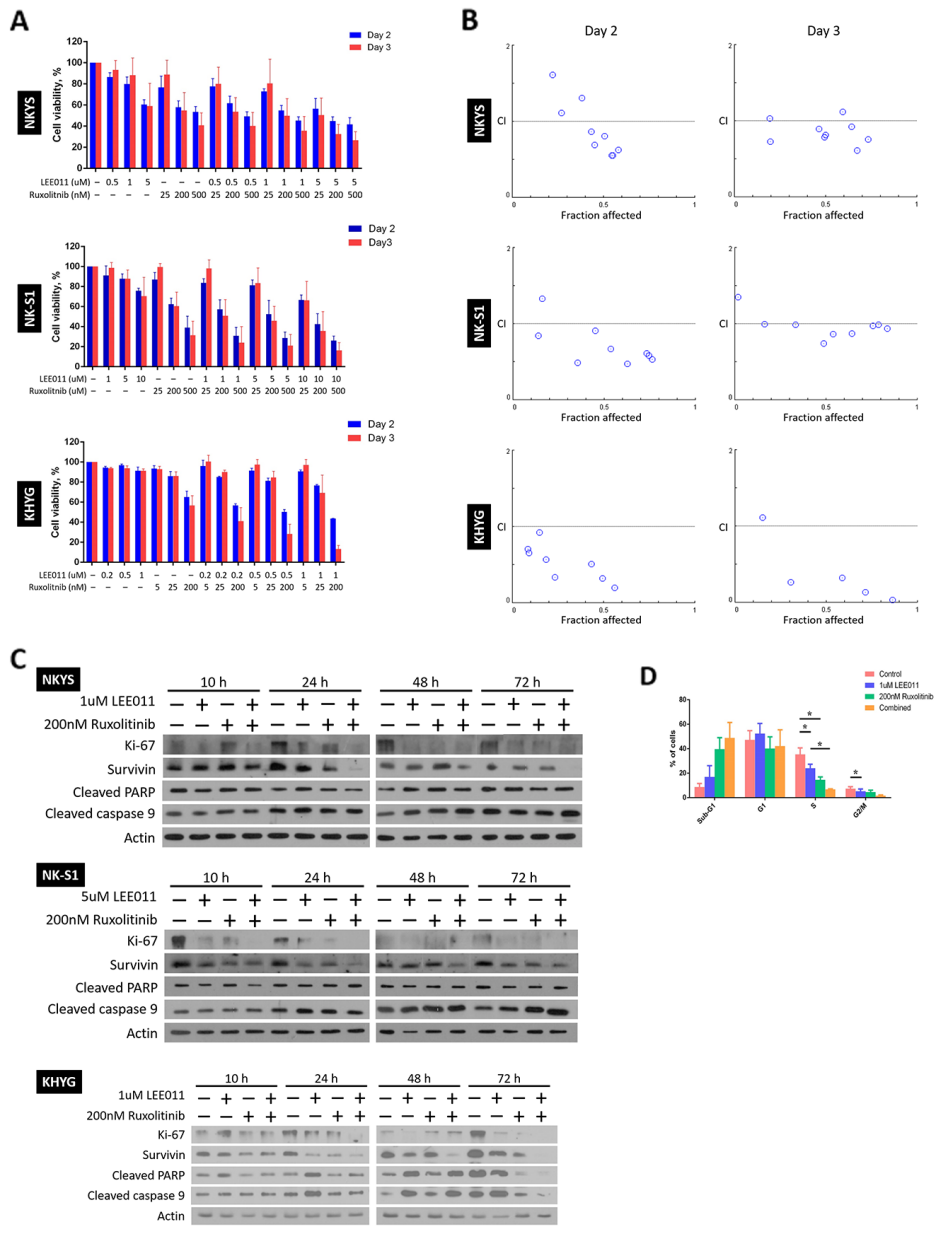
LEE011 and ruxolitinib displayed synergistic relationship on achieving growth inhibition in NKTCL cell lines **(A)** Cell viability assay showed enhanced growth inhibition with dual LEE011 and ruxolitinib treatment. The cells were subjected to single or combined LEE011 treatment as indicated and cell viabilities were assessed on Day 2 and 3. In each experiment, values from triplicate wells were averaged and treatment wells were normalised against control wells. Data is expressed as mean ± SEM from three independent experiments. **(B)** Fa-CI plots for the cells on the days cell viabilities were assessed revealed a synergistic growth inhibition effect with dual treatment of LEE011 and ruxolitinib. In the Fa-CI plots, the dashed line where CI = 1 indicates an additive reaction between the two drugs. Points that fall above (CI>1) and below (CI<1) the dashed line denote antagonism and synergism respectively. **(C)** Western blot analysis of proliferative and apoptotic markers following LEE011 and ruxolitinib combined treatment. Cells were singly and combine treated to LEE011 and ruxolitinib at concentrations as indicated. Protein lysates from 10 and 24 h were ran on the same SDS-PAGE gel, while that of 48 and 72 h were ran on a separate gel. In general, a decrease in proliferative markers and increase in apoptotic markers in combine treatment as compared to single treatment were noted across different time points. **(D)** Evaluation of combination treatment using flow cytometric analysis of cell cycle populations. KHYG cells were treated with LEE011 and ruxolitinib at concentrations as indicated. Significant decrease in S phase populations was noted between single LEE011 and combine treatment (n = 3, ^*^ p<0.05, one-way ANOVA test).

Using the IC50 values of the two drugs determined for each NKTCL cell line, a range of concentrations around the IC50 value was selected for combination treatment. In contrast to the treatment conditions for single treatments, cells were only treated for a maximum period of three days, as any enhanced inhibitory effects should be observed at an earlier time point if LEE011 and ruxolitinib work synergistically with each other. As before, cells were re-treated at Day 2 and cell viabilities were assessed at Day 2 and 3 (Figure 3A). As seen, at a certain concentration of LEE011, a further drop in cell viability was noted when the cells were treated with ruxolitinib as well, and vice versa. These results thus suggest that combining LEE011 and ruxolitinib is able to produce an enhanced growth inhibitory effect in NKTCL cell lines.

However, simply by just noting the further drop in cell viability in combined as compared to single treatment is not sufficient to conclude on the synergism with using LEE011 and ruxolitinib together. To this end, determination of combination indices (CIs) of the two drugs at all combination concentrations was performed. A CI value of more than, equal and smaller than 1 is indicative of antagonism, additive and synergism respectively [26]. As most of the CI values obtained are less than 1, it strongly suggests that there is synergism at most concentrations of the two drugs and the two time points at which cell viabilities were assessed (Figure 3B, refer to Supplementary Table 1 for the numerical CI values).

After noting synergistic growth inhibitory effect of LEE011 and ruxolitinib via cell viability assay, it is then imperative to understand how enhanced growth inhibition is achieved. For this purpose, we studied changes in the expression levels of proliferative and apoptotic markers in the NKTCL cell lines following drug treatment for three days (Figure 3C). Changes in cells’ proliferative capacity were assessed by the levels of the proliferative marker Ki-67 and cell-death inhibitor survivin [27, 28], while cleaved PARP and caspase 9 were probed for any increase in apoptosis [29, 30]. In general, we noticed a decrease in Ki-67 and survivin levels from control to treated cells as early as 10 h following treatment. Importantly, Ki-67 and survivin fell more in combination treatments compared to that of single treatments from around 10 h that persisted up until 72 h, suggesting that the synergistic effect of LEE011 and ruxolitinib occurs at an early time point. As for the apoptotic markers, slightly higher levels of cleaved caspase 9 for all treated cells in contrast to control arose at 24 h. From 48 h, higher levels of cleaved caspase 9 were detected in cells treated with both drugs than cells that received single drug. Cleaved PARP levels remained relatively constant throughout all time points in NKYS and NK-S1 cells, but increased further in combination treatment from 48 h in KHYG cells. These results thus provide promising leads into the mechanism of growth inhibition of LEE011 and ruxolitinib combination treatment.

Beside studying levels of proliferative and apoptotic markers, knowing at which stage of the cell cycle is growth inhibited is also important for us to further understand the mechanism of action of LEE011 and ruxolitinib in NKTCL. Thus, we carried out flow cytometric analysis of cell cycle populations on KHYG after treatment for 48 h (Figure 3D). As seen, there was an increase in sub-G1 population and decrease in both S and G2/M phase populations going from single to combination treatment. Particularly the decrease in S phase between single LEE011 and combination treatment was significant. In summary, this finding further corroborated the synergistic relationship between LEE011 and ruxolitinib, in that inhibiting JAK1/2 on top of CDK4/6 could further prevent entry into S phase of the cell cycle.

## DISCUSSION

The low success rates that current treatments on NKTCL are showing [1, 3, 4] has urged us to discover better treatment alternatives. Several key findings in recent years have helped to shed light on the pathogenesis of NKTCL. In particular, deregulation of JAK-STAT signalling as well as overexpression of cyclin D1 by upregulated EZH2 levels have been noted to confer oncogenic potential in NKTCL [7, 14]. These findings thus suggested for the dual targeting of JAKs and cyclin D1 in NKTCL.

Here, we tested a combination of ruxolitinib and LEE011 that inhibit JAK1/2 and CDK4/6 respectively against several NKTCL cell lines, namely NKYS, KHYG and NK-S1. We first confirmed the growth inhibitory efficacy of separate LEE011 and ruxolitinib treatment via cell viability assays and clonogenic assay. The higher IC50 concentrations noted for LEE011 as compared to ruxolitinib across all NKTCL cell lines tested might suggest a possibility that inhibition of JAK1/2 produces a more potent effect than CDK4/6 inhibition. This might be explained by the coupling of JAKs to the activation of pathways that promote cell survival [10]. Therefore, while LEE011 and ruxolitinib both decrease cell proliferation, ruxolitinib can affect survivability as well.

The effect that LEE011 has on NK-S1 was observably less effective than in NKYS and KHYG, with just about 20% decrease in cell viability at concentrations as high as 10 μM, which is also reflected in a higher IC50 value. This difference in LEE011 growth inhibitory efficacy between the NKTCL cell lines could be due to the mutational status of JAK3 in them. While NKYS and KHYG possess wild-type JAK3, NK-S1 carries a homozygous *JAK3*^*A572V*^ mutation, which makes JAK3 constitutively active [31]. The constitutively active JAK3 could allow for the persistence of pro-survival and pro-proliferation signals in NK-S1 cells, attenuating the effect of LEE011.

Subsequently, it was concluded that a synergistic relationship exists between LEE011 and ruxolitinib as the CI values were less than 1 at most concentrations tested. Still the presence of CI values greater than 1 should not be ignored. In general, a CI value of more than 1 was observed when low concentrations of LEE011 and ruxolitinib were used. However, this does not mean that the two drugs work antagonistically at low concentrations and synergistically at high concentrations. Rather, it is plausible that the growth inhibitory effects obtained at these low concentrations in combination were not very different from that of single treatment. Any increase in cell viability in combination from single treatment at low concentrations could be the result of experimental variations.

Similarly, the strikingly high CI values in KHYG cells (Supplementary Table 1, highlighted in grey) could be due to the same reasons as well. In particular, the 5 nM ruxolitinib dose might have been too low to be included for the assessment of combination effect. This is in view that the IC50 concentrations determined for ruxolitinib at Day 2 and 3 were close to 200 nM, which is 40-fold higher than 5 nM. This could thus account for the high CIs noted at 5 nM ruxolitinib at varying LEE011 concentrations. Considering that CI values were less than 1 in most other instances where sufficiently high concentrations of LEE011 and ruxolitinib were used, it is thus sound to conclude that there is a synergistic effect of inhibiting both CDK4/6 and JAK1/2 in NKTCL.

The synergistic relationship between LEE011 and ruxolitinib was further confirmed by cell cycle analysis, with significant difference going from single to combination treatment noted at S phase. Synergism was also evidenced by changes in proliferation and apoptotic markers, which showed that combination treatment causes further decrease in proliferation and increase in apoptosis. Difference in Ki-67 levels arose as early as 10 h, suggesting that the synergistic effect of LEE011 and ruxolitinib in inhibition of proliferation occurs at an early time point. This was then followed by a loss of the anti-apoptotic molecule survivin at 24 h. The loss of survivin then possibly promoted apoptotic death as seen by an increase in cleaved caspase 9 levels from 48 h onwards. Cell death appears to have occurred via the intrinsic apoptotic pathway, given caspase 9’s exclusive involvement in only this apoptotic pathway [29]. Therefore, the examination of the proliferative and apoptotic markers also revealed the synergistic relationship between LEE011 and ruxolitinib in NKTCL.

The results of LEE011 and ruxolitinib combination treatment thus provided insights into a promising new treatment for NKTCL. The effect of JAK1/2 inhibition with ruxolitinib agrees with other studies where NKTCL growth inhibition was similarly achieved via JAK2 inhibition using AG490 [7, 8]. However, ruxolitinib is favoured here as AG490 is less potent and has been reported to block many other Ser/Thr kinases, which would complicate results interpretation [32]. Our data on ruxolitinib in NKTCL are also in concordance with recent publications [33, 34]. In addition, the combination use of LEE011 and ruxolitinib have been demonstrated to be successful in the treatment of myelofibrosis and is currently undergoing phase I of clinical trials [35, 36]. It is thus hoped that the success of LEE011 and ruxolitinib combination use for myelofibrosis can be leveraged upon to push forward an alternative treatment for NKTCL.

Single ruxolitinib treatment caused no change in EZH2, cyclin D1 and H3K27me3 levels, indicating absence of JAK1/2 regulation on EZH2’s level and activity. However, it is possible that JAK3 is the predominent regulator of EZH2 activity in NKTCL and hence no changes were observed following JAK1/2 inhibition. This is consistent with our previous observation that inhibition of JAK3 with JAK3 inhibitor PF956980 can target deregulated EZH2 pathway using [25].

## MATERIALS AND METHODS

### Cell culture

The cell lines used in this study included NKYS, NK-S1 [37, 38] (both NK lymphoma cell lines and KHYG [39] (NK leukaemia cell line). NKYS and KHYG cells were cultured in Roswell Park Memorial Institution media (RPMI; Hyclone, USA) supplemented with 10% heat-inactivated fetal bovine serum (FBS; Hyclone), 1% each of penicillin-streptomycin (P/S) and L-glutamine (both from Biowest, France). NKYS and KHYG were also supplemented with 20 ng/ml and 40 ng/ml recombinant human interleukin-2 (IL-2; Miltenyi Biotec, Germany) respectively. NK-S1 was cultured in Dulbecco’s Modified Eagles Medium (DMEM; Hyclone) without sodium pyruvate, supplemented with 10% FBS (Hyclone), 10% inactivated horse serum (Life Technologies, USA) and 1% each of P/S and L-glutamine (Biowest). All cell lines were grown in humidified incubators at 37°C with 5% CO_2_.

### Drugs

Drugs used in this study included the CDK4/6 inhibitor, LEE011 (Novartis, Switzerland), and the JAK1/2 inhibitor, ruxolitinib (Invivogen, USA).

### Cell viability assay

Cells were seeded at 2 × 10^4^ cells/100μl/well in 96-well plates in triplicates and treated with or without the respective drugs at various concentrations for a maximum period of four days. The cells were re-treated at Day 2 to account for loss of drug stability in the incubator over extended time period. Cell viability was assessed at Day 2, 3 and 4 using the CellTiter-Glo 2.0 assay (Promega, USA) as per manufacturer’s protocol and luminescence was read using the Infinite M200 plate reader (Tecan, Switzerland).

### Protein concentration estimation

Cells were lysed in radioimmunoprecipitation assay buffer and were subjected to sonication. Protein lysates were 10-fold diluted and standards were prepared by serial dilution of 1 mg/ml bovine serum albumin (BSA; Hyclone). Standards and samples were all done in triplicates. 200 μl of Quick Start™ Bradford reagent (Bio-Rad, USA) was then added and the intensity of the resulting blue colour was measured at a wavelength of 595 nm.

### Western blotting

Appropriate percentage of sodium dodecyl sulfate-polyacrylamide gel electrophoresis (SDS-PAGE) was used to resolve equal amounts of protein samples. The resolved protein samples were then transferred to polyvinylidene difluoride membrane (Bio-Rad). After which, the membrane was blocked in 5% BSA solution and incubated overnight with primary antibodies at 4°C, followed by incubation for 1 hour with secondary antibodies at room temperature. Primary antibodies used included BioLegend antibody Ki-67 (652402), Cell Signalling antibody cyclin D1 (2978), cleaved caspase 9 (9501), cleaved poly(ADP-ribose) polymerase (PARP; 9541), EZH2 (3147), H3 (9715), H3K27me3 (9733, 9756), p-STAT3 (9131, sc-8059), p-STAT5 (9351, 9356), survivin (2802), and SantaCruz antibody actin (sc-1615HRP), CDK4 (sc-23896), CDK6 (sc-7961), GAPDH (sc-47724 HRP), STAT3 (sc-482), STAT5 (sc-835). Secondary antibodies used included SantaCruz antibody goat anti-rabbit IgG-HRP (sc-2030) and goat anti-mouse IgG-HRP (sc-2031). Blotted proteins were detected and visualised using Amersham™ ECL™ start Western Blotting detection reagent (GE Healthcare, UK), Luminata™ Crescendo Western HRP substrate (Millipore, USA) or SuperSignal™ West Femto Maximum Sensitivity Substrate (Thermo Scientific, USA). Where necessary, p-STAT3 and p-STAT5 antibodies were stripped using Restore™ Western Blot Stripping Buffer (Thermo Scientific) following manufacturer’s recommendations to re-probe for STAT3 and STAT5 respectively.

### Clonogenic assay

5000 NKYS, KHYG and NK-S1 cells were seeded separately into each well of a 6-well plate in StemMACS hematopoietic stem cell-colony forming units (HSC-CFU) basic methylcellulose medium (Miltenyi Biotec). Appropriate concentration of IL-2, LEE011 and ruxolitinib were also added into each well. The plates were placed in humidified incubators at 37°C with 5% CO_2_ for two weeks, after which colony formation was scored with an inverted microscope.

### Plasmid and siRNA transfection

Transfection on NKTCL cell lines were done using Neon^®^ transfection system (Invitrogen) following manufacturer’s protocol at the electroporation parameters of 1250V, 10 ms width and three pulses. For each transfection, 1 × 10^6^ cells were used. For overexpression experiment, NKYS cells were transfected with 2 μg control (pCMV-HA-GFP) or EZH2 (pCMV-HA-EZH2WT; kindly gifted by Dr Yu Qiang of GIS) vector plasmid, 0.5 μg green fluorescence protein plasmid (pMAX-GFP) and 0.5 μg puromycin-resistant plasmid. For knockdown experiment, 200 nM human non-targeting siRNA negative control #1 (4390844) or 100 nM each of siRNA against CDK4 (s2822) and CDK6 (s51) were used and were all purchased from Life Technology Ambion.

### Quantitative RT-PCR

Cells were lysed using QIAzol lysis reagent (Qiagen, Germany) and total RNA was isolated using the RNeasy Mini Kit (Qiagen). The extracted RNA was then converted to cDNA with the iScript Reverse Transcription Supermix (Bio-Rad). The mRNA levels of *EZH2*, *CCND1* and housekeeping gene *GAPDH* were then quantitated using iTaq™ Universal SYBR^®^ Green Supermix (Bio-Rad) according to manufacturer’s recommendations, using their respective primers (Supplementary Table 2).

### Flow cytometric assay for cell cycle

To measure the effect of LEE011 and ruxolitinib combinatorial treatment on apoptosis, NKYS, KHYG and NK-S1 were separately treated at 2 × 10^5^ cells/ml in 10 cm dishes at appropriate drug concentrations, and harvested 48 h later. Cells were washed and fixed in ice-cold 70% ethanol for 2 h before further incubation at -20°C for up to 24 h. Immediately before flow cytometric analysis, the cells were treated with RNase A (Qiagen) and stained with propidium iodide (Invitrogen, USA) for 30 min. Collection and analysis of data was done using BD LSR II Special Order System (BD Biosciences, USA).

### Statistical analysis

All graphs are expressed as mean ± SEM, from three independent experiments. Difference between samples were evaluated by unpaired one-tailed student’s *t*-test or one-way ANOVA test where appropriate, and p-values < 0.05 were considered as statistically significant. IC50 curves (variable slope) were plotted using GraphPad Prism 6.1 (GraphPad Software, USA) and IC50 values over different treatment time points were compared via the extra sum-of-squares F test. Synergistic effects from drug combination was determined via the quantitative method using CompuSyn [26] (version 1.0, ComboSyn, Inc.).

## SUPPLEMENTARY MATERIALS FIGURES AND TABLES


