# Supplementary material for: LEE011 and ruxolitinib: a synergistic drug combination for natural killer/T-cell lymphoma (NKTCL)
Source: Oncotarget. 2018 Aug 7;9(61):31832–41. doi: 10.18632/oncotarget.25835 (PMC6112754; doi:10.18632/oncotarget.25835)
Supplement: Supplementary file 1 [file oncotarget-09-31832-s001.pdf]

LEE011 and ruxolitinib: a synergistic drug combination for natural killer/T-cell lymphoma (NKTCL)

SUPPLEMENTARY MATERIALS

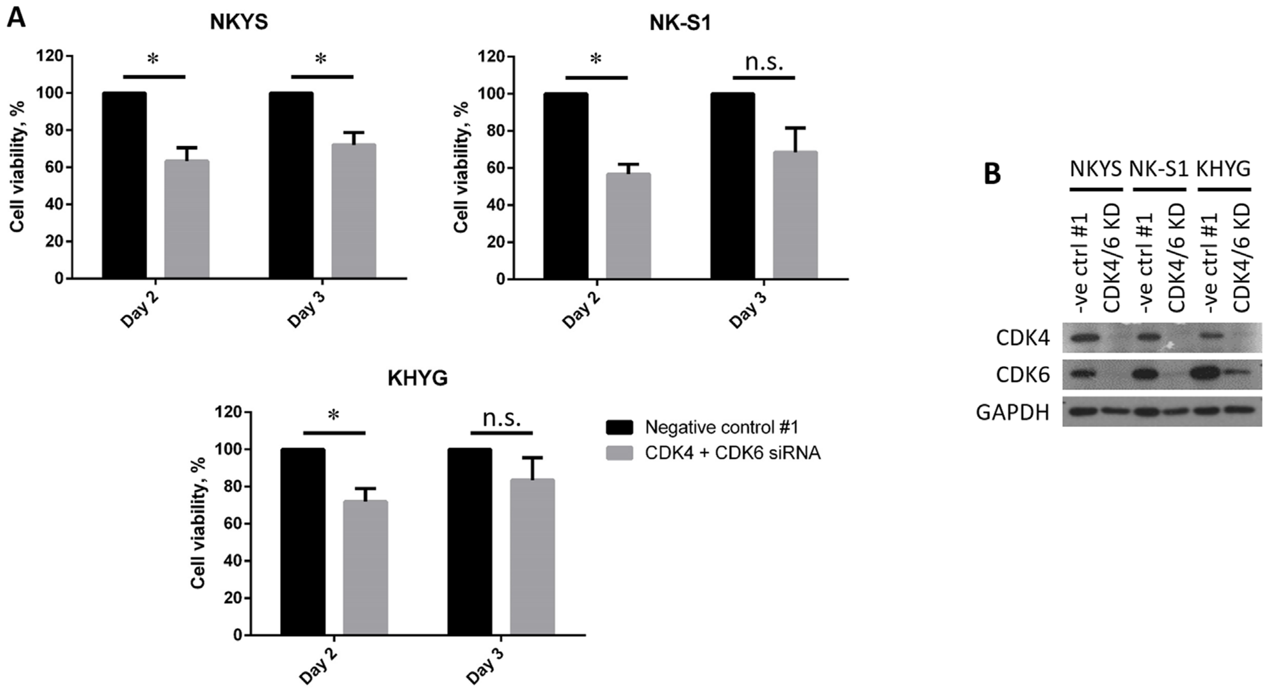

Supplementary Figure 1: CDK4/6 siRNA KD inhibits growth in NKTCL cell lines. (A) Cell viability assay showed growth inhibition following dual KD of CDK4 and CDK6 compared to negative control #1 siRNA. In each experiment, duplicate values were averaged and cells that received CDK4/6 siRNA were normalised against those that received the control siRNA. Data is expressed as mean ± SEM from three independent experiments (n = 3, * $p<0.05$, one-tailed student's *t*-test). **(B)** Western blot analysis of the KD efficiency of CDK4 and CDK6. Cells were harvested on Day 2 after transfection.

Supplementary Table 1: CI values for different combination concentrations of LEE011 and ruxolitinib for (A) NKYS, (B) NK-S1 and (C) KHYG

A

**LEE011/uM**	**0.5**			**1**			**5**		
**Ruxolitinib/nM**	**25**	**200**	**500**	**25**	**200**	**500**	**25**	**200**	**500**
**Day 2**	1.61	1.31	0.81	1.11	0.69	0.56	0.87	0.55	0.63
**Day 3**	0.73	0.90	1.12	1.03	0.82	0.92	0.79	0.61	0.76

B

**LEE011/uM**	**1**			**5**			**10**		
**Ruxolitinib/nM**	**25**	**200**	**500**	**25**	**200**	**500**	**25**	**200**	**500**
**Day 2**	0.84	0.91	0.62	1.33	0.67	0.58	0.49	0.47	0.54
**Day 3**	1.35	0.74	0.98	0.99	0.87	0.99	0.99	0.88	0.94

C

**LEE011/uM**	**0.2**			**0.5**			**1**		
**Ruxolitinib/nM**	**5**	**25**	**200**	**5**	**25**	**200**	**5**	**25**	**200**
**Day 2**	4.39	0.93	0.51	0.71	0.57	0.33	0.66	0.34	0.20
**Day 3**	29.35	2.44	0.33	175.27	1.11	0.13	167.32	0.26	0.03

Fraction inhibition at each condition was determined from the cell viability assay after averaging effects over three independent experiments. CI values of synergistic (CI < 1), additive effect (CI = 1) or antagonistic (CI > 1) relationship between two or more drugs.

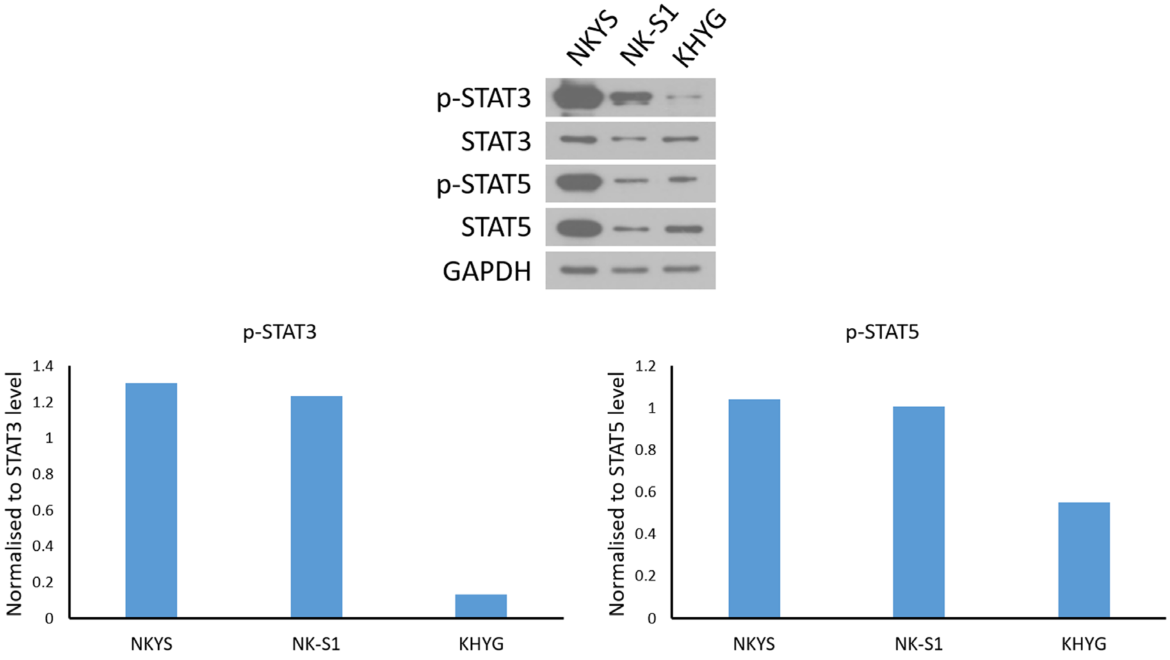

Supplementary Figure 2: Basal phosphorylation of STAT3/5 proteins in NKYS, NK-S1 and KHYG cell lines. Cells were harvested two days after subculture. Phosphorylated STAT3/5 levels in each cell line were normalised with their respective total STAT3/5 levels to enable comparison between cell lines.

Supplementary Table 2: Primer sequences for quantitative RT-PCR analyses

Gene	Direction	Sequence
CCND1	F	GCTCCTGGTGAACAAGCTCAA
	R	TTGGAGAGGAAGTGTTCAATGAAA
EZH2	F	AGGAGTTTGCTGCTGCTCTC
	R	CCGAGAATTTGCTTCAGAGG
GAPDH	F	GGCTGTGGGCAAGGTCATCCCTGAG
	R	GTCGCTGTTGAAGTCAGAGGAGACCACCTG

F and R stands for forward and reverse primers respectively.
